# Identification and characterization of a *Drosophila* ortholog of WRN exonuclease that is required to maintain genome integrity

**DOI:** 10.1111/j.1474-9726.2008.00388.x

**Published:** 2008-06

**Authors:** Robert D C Saunders, Ivan Boubriak, David J Clancy, Lynne S Cox

**Affiliations:** 1 Department of Biological Sciences, The Open University Milton Keynes MK7 6AA, UK; 2 Department of Biochemistry, University of Oxford Oxford OX1 3QU, UK

**Keywords:** exonuclease, *Drosophila*, genome stability, homologous recombination, Werner syndrome, WRN

## Abstract

The premature human aging Werner syndrome (WS) is caused by mutation of the RecQ-family WRN helicase, which is unique in possessing also 3′–5′ exonuclease activity. WS patients show significant genomic instability with elevated cancer incidence. WRN is implicated in restraining illegitimate recombination, especially during DNA replication. Here we identify a *Drosophila* ortholog of the WRN exonuclease encoded by the *CG7670* locus. The predicted DmWRNexo protein shows conservation of structural motifs and key catalytic residues with human WRN exonuclease, but entirely lacks a helicase domain. Insertion of a piggyBac element into the 5′ UTR of *CG7670* severely reduces gene expression. DmWRNexo mutant flies homozygous for this insertional allele of *CG7670* are thus severely hypomorphic; although adults show no gross morphological abnormalities, females are sterile. Like human WS cells, we show that the DmWRNexo mutant flies are hypersensitive to the topoisomerase I inhibitor camptothecin. Furthermore, these mutant flies show highly elevated rates of mitotic DNA recombination resulting from excessive reciprocal exchange. This study identifies a novel WRN ortholog in flies and demonstrates an important role for WRN exonuclease in maintaining genome stability.

## Introduction

Werner syndrome (WS) provides a very useful model system for the study of human aging at the molecular level, with patients manifesting many signs of normal aging in an accelerated manner (reviewed in Martin, 1985; Goto, 2001; Cox & Faragher, 2007; Kudlow *et al*., 2007). The syndrome is caused by mutation of WRN (Yu *et al*., 1996), a member of the RecQ DNA helicase family. WS patient-derived cells undergo highly premature replicative senescence, with cellular defects including aberrant DNA replication (Pichierri *et al*., 2001; Rodriguez-Lopez *et al*., 2002) and hyper-recombination (Salk *et al*., 1981; Scappaticci *et al*., 1982; Fukuchi *et al*., 1985). Hypersensitivity to the DNA-damaging agent 4-nitroquinoline oxide, and the topoisomerase I inhibitor camptothecin (CPT) is characteristic of WS cells (Poot *et al*., 1999; Prince *et al*., 1999; Pichierri *et al*., 2000b). These agents lead to replication fork arrest or collapse, suggesting a function for WRN in DNA replication, which is further supported by its presence at replication foci coincident with RP-A and PCNA (Constantinou *et al*., 2000; Rodriguez-Lopez *et al*., 2003) and aberrant replication fork progression in WS fibroblasts (Rodriguez-Lopez *et al*., 2002). The hyper-recombinant phenotype of human WS cells, suppression of illegitimate recombination in yeast *Sgs1* mutants by human WRN (Yamagata *et al*., 1998), interaction with MRN on replication fork stalling (Franchitto & Pichierri, 2004), the recovery of proliferative capacity after ectopic expression of a Holliday junction resolvase in WS cells (Rodriguez-Lopez *et al*., 2002), and excessive chromosome breakage at fragile sites in the absence of WRN (Pirzio *et al*., 2008) all suggest an important role for WRN in homologous recombination after replication fork arrest, either in preventing the formation of homologous recombination intermediates or in their rapid resolution (Dhillon *et al*., 2007; Rodriguez-Lopez *et al*., 2007). The importance of WRN in regulating genome stability is highlighted by the high cancer incidence in WS patients, while epigenetic inactivation of WRN is also associated with human cancer (Agrelo *et al*., 2006).

Identification of the action of WRN in homologous recombination is complicated by the presence of two enzymatic activities within the same protein: the 3′–5′ helicase characteristic of all RecQ family members (reviewed in Bachrati & Hickson, 2003), and a 3′–5′ exonuclease activity (Huang *et al*., 1998) unique within this family, but which is closely related structurally to the DnaQ exonuclease superfamily (Perry *et al*., 2006). X-ray crystallographic analysis of the exonuclease domain of human WRN suggests a role in DNA end processing (Perry *et al*., 2006), possibly at the stage of strand resection after double-strand breaks, such breaks that as occur at collapsed replication forks. This would be consistent with the importance of WRN in DNA recombination. However, the closely related BLM helicase can, at least *in vitro*, promote dissolution of double Holliday junctions without intrinsic nuclease activity (Wu & Hickson, 2003); determining the relative contributions to homologous recombination of the helicase and exonuclease activities of WRN is therefore important. Moreover, there appears to be a complex interplay between the helicase and exonuclease activities of WRN (Opresko *et al*., 2001); for example, it is possible that helicase activity may be required to generate a template suitable for cleavage by the nuclease. The distinct roles are difficult to dissect in vertebrate cells since ablation of one activity may affect the other; indeed, point mutation of the helicase is suggested to act in a dominant negative manner (Crabbe *et al*., 2004). RNAi depletion of WRN, although highly effective in recapitulating some WS-like phenotypes (Dhillon *et al*., 2007), eliminates both helicase and exonuclease activities.

To study WRN's role in recombination at the organismal level, we sought to develop a model in which WRN activity may be evaluated at different developmental stages. Although murine models have been described which show some WS-like features on mutation of the WRN helicase alone or with co-mutation of either telomerase or PARP (Lebel, 2002; Lebel *et al*., 2003; Chang *et al*., 2004; Massip *et al*., 2006), the relatively long lifespan and complexity of genetic intervention pose severe limitations on their exploitation. In order to develop a model system more amenable to genetic and biochemical analysis of WRN exonuclease function *in vivo*, we set out to identify and characterise WRN exonuclease from *Drosophila melanogaster*.

## Results

### Identification of *Drosophila* WRNexo

We conducted a BLASTP search (Altschul *et al*., 1997) of the *Drosophila melanogaster* genome sequence (Release 4.0), using as a probe the sequence of human WRN protein. The *Drosophila* candidate gene encoding a WRN-like exonuclease is *CG7670*, with an E-value of 1 × 10^−25^ (Cox *et al*., 2007, as also noted by Sekelsky *et al*., 2000). Upon cloning from mRNA and sequencing multiple *CG7670* cDNA clones, we found two alleles of CG7670 differing solely by the presence or absence of an AAG codon (lysine) at nucleotide 235, amino acid 79 (GenBank accession numbers EF680279 and EF680280, respectively). Variant 2 (EF680280) lacking lysine 79 was the more commonly occurring clone, encoding a predicted protein of 352 amino acids.

**Fig. 1:**
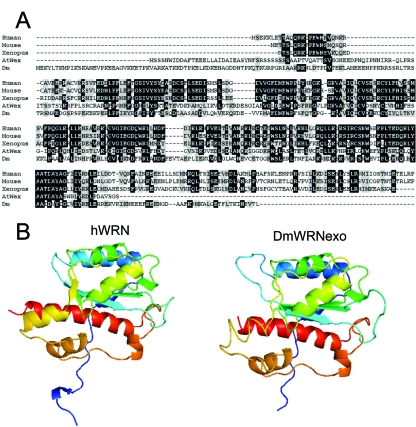
Homology of DmWRNexo to human WRN. (A) Alignment of WRN protein sequences from human, mouse, *Xenopus laevis* and *Arabidopsis thaliana* (AtWEX) with full-length DmWRNexo (predicted from both our cloned *CG7670* cDNA and genomic sequence (Flybase)). Black depicts residues identical in three or more species, with grey showing similarity. Note the highly conserved blocks of sequence flanking the catalytic core residues. D82 and E84 metal-ion co-coordinating residues (residues numbered for the human WRN protein) are marked with an asterisk. (B) SWISS-MODEL was used to predict the possible tertiary structure of DmWRNexo from residues 118–312, according to the known structure of human WRN exonuclease (2fbyA, Perry *et al*., 2006). Left panel shows the human WRN exonuclease domain while the right panel shows the predicted structure of *Drosophila melanogaster* DmWRNexo.

The predicted protein product of the *CG7670* locus, which we call DmWRNexo, shares 35% identical and 59% similar amino acids with the exonuclease domain of human WRN over a region of 192 residues (Fig. 1A). Previous crystallographic studies of the human WRN exonuclease domain demonstrated that residues aspartate (D82) and glutamate (E84) within the nuclease catalytic site are essential for metal ion coordination (Perry *et al*., 2006). Importantly, these residues are conserved in DmWRNexo (Fig. 1A, asterisks). We have conducted SWISS-MODEL structural predictions (Peitsch, 1996; Schwede *et al*., 2003) of DmWRNexo from residues 118–312, which suggest that the protein might adopt a very similar configuration to human WRN exonuclease (Perry *et al*., 2006, PDB accession number 2fbyA, predicted similarity e-value 8.86 × 10^−26^), with conservation of key alpha helices and beta sheets comprising the nuclease active site (Fig. 1B).

### Hypomorphic allele of CG7670

**Fig. 2:**
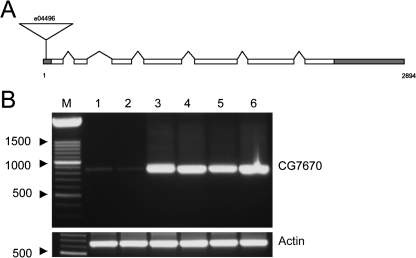
*CG7670^e04496^* mutant is severely hypomorphic. (A) The predicted structure of the *CG7670* transcript. The location of the piggyBac insertion e04496 is indicated. (B) Reverse transcription–polymerase chain reaction (RT-PCR) indicates that *CG7670* is expressed at very low levels compared with heterozygotes or wild-type controls. cDNA was generated by one-step RT-PCR using primers specific to correctly spliced *CG7670*(upper panel) or actin 5C (lower panel), yielding products of 854 bp (*CG7670*) and 652 bp (actin). Lanes 1, 2 *CG7670^e04496^* homozygotes; lanes 3, 4 *CG7670^e04496^* heterozygotes; lanes 5, 6 wild type. Lanes 1, 3, 5 = male; lanes 2, 4, 6 = female.

To assess the impact of DmWRNexo mutation on flies, we obtained an insertional mutant allele of *CG7670*, *CG7670^e04496^*, which contains a piggyBac{RB} element (Thibault *et al*., 2004) inserted within the 5′ UTR (Fig. 2A). Reverse transcription–polymerase chain reaction (RT-PCR) analysis shows that the *CG7670^e04496^* allele is transcribed at an extremely low level in the homozygous mutant compared with *CG7670* expression in heterozygous and wild-type flies (Fig. 2B); no band was detectable in negative controls (data not shown). Interestingly, *CG7670^e04496^* homozygotes show no gross morphological abnormality, and while the females are sterile (eggs do not hatch), males are fertile. The location of *CG7670* on chromosome 3R:14189966.14191859 (Flybase) and its identity with the gene encoding DmWRNexo is consistent with our mitotic recombination deficiency mapping studies (*mwh^1^ CG7670^e04496^*/Df(3R)Exel6178 flies display multiple wing hair clones; data not shown).

### DmWRNexo mutant flies are hypersensitive to CPT

**Fig. 3:**
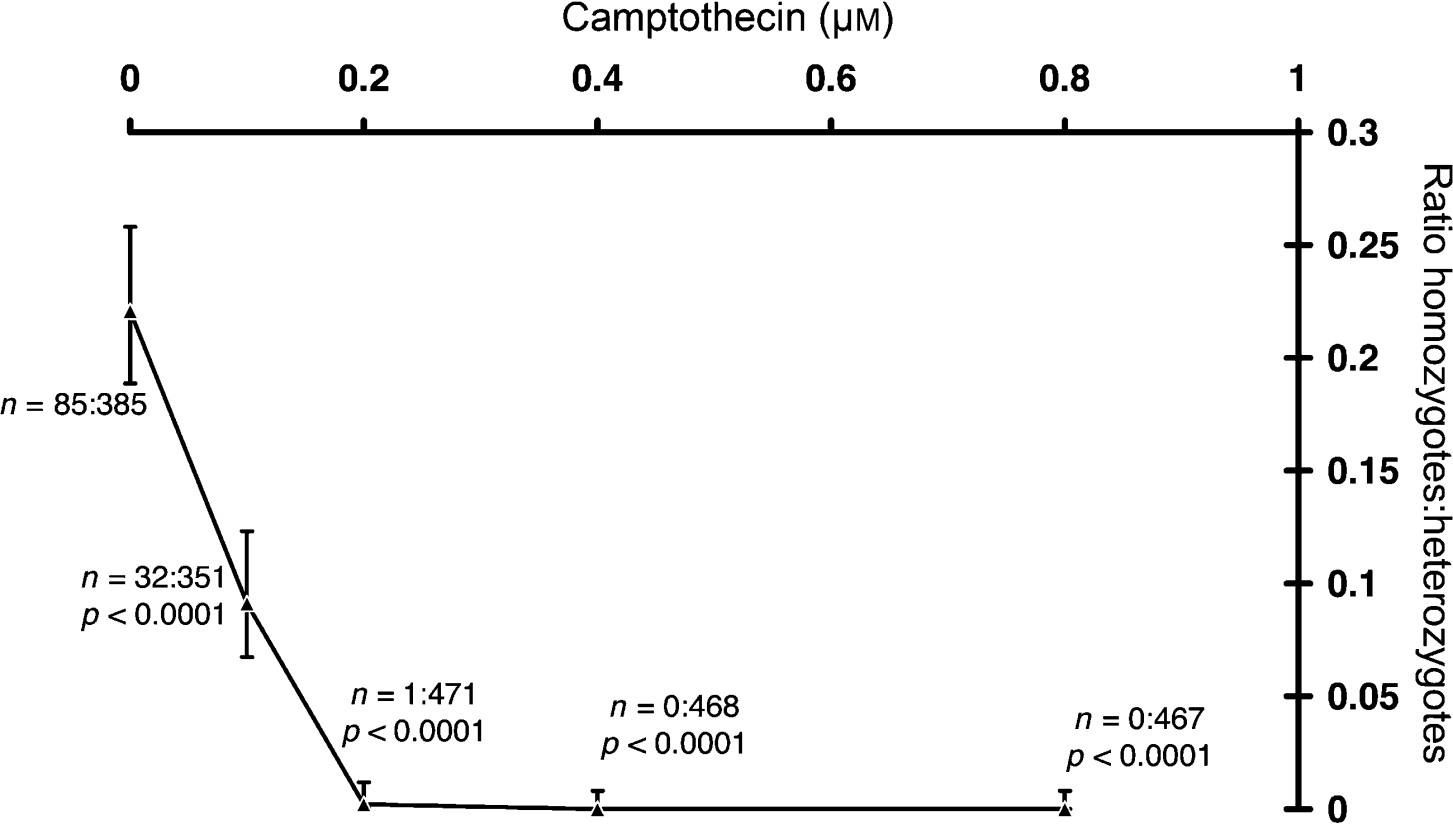
DmWRNexo mutant flies are sensitive to camptothecin (CPT). Flies heterozygous for the *CG7670^e04496^* allele were crossed and progeny reared on medium supplemented with various concentrations of CPT. The proportion of all emerging adult flies that were homozygous for *CG7670^e04496^* was plotted against CPT concentration; heterozygous flies were fully viable at all CPT concentrations tested. The lower frequency of homozygous flies observed at increasing drug doses demonstrates hypersensitivity to CPT. *n* represents the ratio of *CG7670^e04496^* homozygous: heterozygous flies. *p*-values refer to the Fisher exact test for the probability that the proportion of homozygotes was less than that of the control value (0 µm CPT). The error bars show binomial 95% confidence intervals.

Werner syndrome cell lines are sensitive to the topoisomerase I poison CPT (Poot *et al*., 1999; Pichierri *et al*., 2000a) which causes replication fork collapse at the bound topoisomerase (Shao *et al*., 1999); such sensitivity can be partially complemented by expression of a bacterial Holliday junction nuclease (Rodriguez-Lopez *et al*., 2007), suggesting that WRN acts either to prevent accumulation of Holliday junctions at collapsed forks or to ensure rapid Holliday junction resolution. To test whether *Drosophila* mutant for *DmWRNexo* are similarly sensitive to CPT, larvae derived from crosses of *CG7670^e04496^* heterozygotes were propagated on medium supplemented with varying concentrations of CPT, or vehicle-only control (0 µm CPT). Emerging flies were scored for heterozygosity or homozygosity of the *CG7670^e04496^* allele. While the heterozygous flies appear to be fully viable at all concentrations of CPT used (Fig. 3), a significant loss of viability of flies homozygous for the *CG7670^e04496^* allele (i.e. those with very low levels of expression of DmWRNexo) was observed even at 0.1 µm CPT, with almost total lethality from 0.2 µm (Fig. 3). Surviving homozygotes displayed roughened eyes, an indicator of cell death, and many died as pharate adults (data not shown), a typical lethal phase for flies exhibiting high levels of cell death. This is consistent with the high levels of apoptosis detected in human Werner syndrome cells exposed to CPT (Poot *et al*., 1999). Thus, loss of DmWRNexo results in hypersensitivity to CPT.

### Genome instability in DmWRNexo mutant flies

Since hyper-recombination is a key phenotype of WS patient-derived cell lines (Salk *et al*., 1981; Scappaticci *et al*., 1982; Fukuchi *et al*., 1985), rates of chromosome breakage and/or mitotic recombination in *DmWRNexo* homozygous mutant flies were evaluated using the recessive multiple wing hairs marker (*mwh*, recombination map position 3-0.7); wing blade cells hemizygous or homozygous for *mwh^1^* develop tufts of wing hairs instead of single hairs. Note that the adult wing consists of postmitotic cells arising from proliferating cells of the wing imaginal disc, so any recombination giving rise to clones of cells with the mwh phenotype must have occurred during cell proliferation in development.

**Fig. 4:**
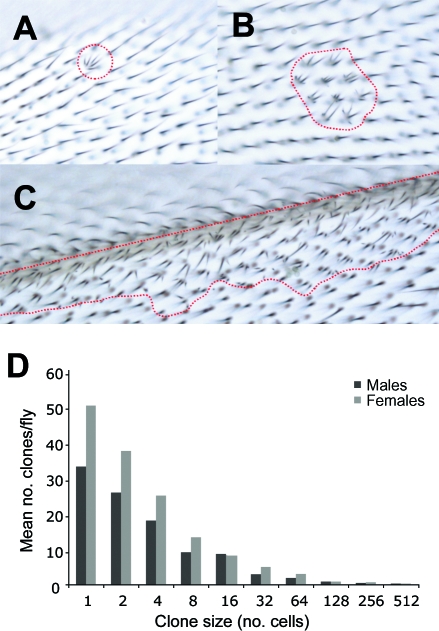
Hyper-recombination in *DmWRNexo* mutant flies. (A–C): Wing blades of flies homozygous for the hypomorphic allele of *DmWRNexo* (*w*; *mwh^1^ CG7670^e04496^*/*CG7670^e04496^*) were analysed microscopically; mwh clones are outlined with a broken line. (A) single-cell clone, (B) 10-cell clone, and (C) moderate-sized clone of mwh cells. (D) Frequency of mwh clones in wing blades of DmWRNexo homozygous mutant flies (*w*; *mwh^1^ CG7670^e04496^*/*CG7670^e04496^*) plotted against clone size, i.e. number of cells comprising the clone (number of clones counted: males *n* = 1278, females *n* = 1584). mwh clones were infrequent (0.2 per fly) in heterozygous controls, and all were single-cell clones (not shown).

Wing blades were dissected from flies that were homozygous mutant for *DmWRNexo* but heterozygous for *mwh* (i.e. *w^1118^*; *mwh^1^ CG7670^e04496^*/*CG7670^e04496^*) and analysed microscopically (Fig. 4A–C). The frequency and size of clones showing multiple wing hairs was determined (Fig. 4D), demonstrating that *mwh* clones occur at a very high frequency in the *DmWRNexo* homozygous mutant flies, with an average of over 100 clones per fly. This is in sharp contrast to flies heterozygous for *DmWRNexo* which show a mean of 0.2 *mwh* clones per fly (data not shown). Furthermore, some *mwh* clones in *DmWRNexo* homozygous mutant wing blades were very large (> 500 cells) (Fig. 4D), while the rare clones observed in heterozygous flies were all single cells (data not shown). In addition to the very high rates of recombination detected in *DmWRNexo* mutant flies, these data also demonstrate that the *CG7670^e04496^* allele is recessive, as reported for patient-derived human WRN mutations (Yu *et al*., 1996; Moser *et al*., 1999).

**Fig. 5:**
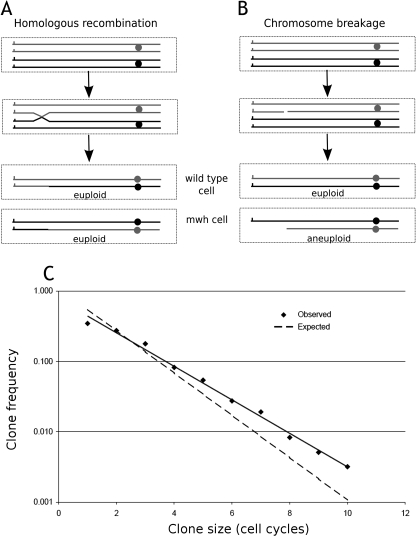
Reciprocal exchange is the major mechanism of mwh clone origin. (A, B) Recombinational origin of mwh clones. Flies are initially heterozygous for *mwh^1^* and homozygous for *CG7670^e04496^*. The parental chromosomes (shown as two sister chromatids linked at the centromere) are depicted in grey (*mwh^+^*) and black (*mwh^1^*); the *mwh* locus is indicated by a tick mark. (A) Homologous recombination between *mwh* and the centromere gives rise to euploid *mwh^+^*/*mwh^+^* and *mwh^1^*/*mwh^1^* daughter cells. (B) Chromosome breakage between *mwh^+^* and the centromere gives rise to one euploid *mwh^1^*/*mwh^+^* daughter cell of wild-type phenotype (and so not scored) and one aneuploid daughter cell that is hemizygous for the mutant *mwh^1^* allele, and which therefore shows the mwh phenotype. (C) The proliferation rate of *mwh* clones supports mitotic exchange as the principal cause of clone formation. Logarithmic plot of the frequency of clones of each clone size class against clone size, where clone size is expressed in numbers of cell cycles completed. If recombination results in euploid cells which proliferate at a normal rate, the expectation is that clones of size *n* cell cycles should be twice as frequent as clones of cell cycle *n* + 1. This results in a line indicated as ‘Expected’ (broken line) in the graph. Should the causative mechanism result in slowly growing cells (for example, if the marked clones are derived from aneuploid cells), the distribution of clone frequencies would tend towards a line of steeper gradient. The gradient of the observed distribution (diamonds) corresponds well to the gradient expected (broken line) for euploid cells (see text for further details).

Cells in the wing blade showing the recessive multiple wing hairs phenotype could genetically be either homozygous or hemizygous for the *mwh^1^* allele. Homozygous *mwh^1^* cells would result from mitotic recombination via a reciprocal exchange event (Fig. 5A); daughter cells should be euploid without any loss of proliferative fitness. By contrast, chromosome loss or single chromosome/chromatid breakage events (Fig. 5B) would give rise to segmentally aneuploid hemizygous *mwh* cells, which would be predicted to proliferate more slowly than euploid cells. To distinguish between these possibilities, we have measured the size of each clone in terms of the number of cell cycles since its generation, and plotted the proportion of clones in each size class (on a logarithmic scale) against clone size (Fig. 5C). This type of analysis informs on the nature of the event leading to clone formation as the gradient of the plot reflects the growth rate of the clones (Baker *et al*., 1978). If clones proliferate at the same rate as surrounding normal cells, the gradient will closely parallel the expectation (that clones of size class *n* will be twice as numerous as clones of size class *n* + 1). Clones that proliferate more slowly, as would be expected for segmental aneuploids, would fit a line of steeper gradient. Our results (‘Observed’, Fig. 5C) indicate that cells comprising wing blade clones in *DmWRNexo* mutant flies proliferate essentially as expected for euploid cells. However, the gradient is slightly shallower than expected for two reasons. First, adjacent smaller clones may have been scored as a single larger clone, and second, the recombination events happening earlier in the lineage of the clone (yielding large clones) depletes the pool of cells from which later events (smaller clones) can occur. The actual frequency of recombination events occurring may also be higher than that observed, since sister chromatid exchange is not scored in this assay. We therefore propose that mitotic recombination is the predominant cause of *mwh* clones in these flies. Based on mathematical simulations (data not shown), we estimate the recombination frequency on chromosome arm 3L to be at least 0.01 event per cell division. Assuming a similar frequency throughout the genome, this corresponds to an overall frequency of at least 0.05 recombination events per cell division.

**Fig. 6:**
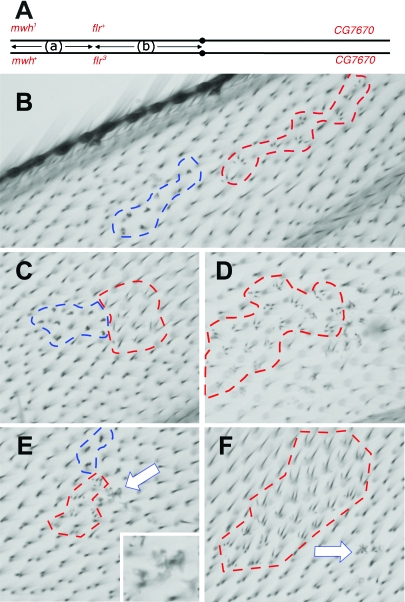
Twin-spot analysis confirms that hyper-recombination occurs through reciprocal exchange. (A) Position of *mwh^1^* and *flr^3^* markers relative to the centromere and *CG7670* locus on chromosome 3 (not to scale). (B–F) Wing blade twin-spot *mwh flr* clones were analysed microscopically. *mwh* clones are outlined with red broken lines and *flr* clones by blue broken lines. (B, C) *mwh-flr* twin spots resulting from recombination proximal to *flr* (within interval (b) in diagram). (D–F) complex clones indicating sequential recombination events. Arrows in (E), (F) and the inset in (E) indicate a possible *mwh flr* double mutant cell.

To further distinguish between chromosome breakage and homologous recombination as the principal mechanism for the elevated frequency of wing blade clones in *CG7670^e04496^* homozygotes, we used a second cuticular marker, *flare* (*flr*, recombination map position 3–38.8), which causes wing blade hairs to be malformed. Wing blades of flies mutant for DmWRNexo and *trans*-heterozygous for *mwh^1^* and *flr^3^* (*w^1118^*; *mwh^1^ CG7670^e04496^*/*flr^3^ CG7670^e04496^*) were examined for *mwh* clones (arising as a consequence of recombination in the chromosomal interval between *mwh* and *flr*) and neighbouring *mwh* and *flr* clones (twin spots), resulting from recombination proximal to *flr* (Fig. 6). If the primary mechanism of genome instability in *DmWRNexo* mutants is chromosome breakage, *mwh flr* twin spots should be rare, while if mitotic recombination is the principal cause of wing blade clones, twin spots will be frequent. The observation of *mwh flr* twin spots at high frequency (Fig. 6E,F) strongly suggests that wing blade clones in the *DmWRNexo* mutants arise predominantly through mitotic recombination. Because of the high frequency of recombination in *CG7670^e04496^* homozygotes, numerical analysis of the twin-spot frequency is difficult and further compounded by sequential repeated recombination events in clone lineages, as inferred from the observation of putative *mwh flr* double mutant cells (Fig. 6E,F). Nonetheless, it is clear that a significant proportion of wing blade clones are derived from homologous exchange. This is in marked contrast to the high rates of chromosomal breakage and loss observed in flies mutant for the RecQ homolog DmBLM (*mus309*) (Kusano *et al*., 2001). Additionally *CG7670^e04496^* homozygote males are fully fertile, inconsistent with chromosomal breakage.

## Discussion

We have identified the locus *CG7670* as the *Drosophila* ortholog of human WRN exonuclease. The encoded DmWRNexo protein shows significant structural and sequence similarities to human WRN exonuclease domain, and moreover, a severe hypomorphic mutation of the locus results in both hyper-recombination and CPT hypersensitivity in flies, features characteristic of human WS cells.

Our data demonstrate that loss of DmWRNexo function leads to very high levels of recombination in the developing *Drosophila* wing (and presumably also in other dividing tissues), consistent with hyper-recombination reported in cells from WS patients (Salk *et al*., 1981; Scappaticci *et al*., 1982; Fukuchi *et al*., 1985). The high frequency of twin spots in wing blade clones seen here strongly supports the assertion that the majority of marked clones in the mutant flies arise as a result of homologous recombination rather than chromosomal breakage. We cannot at this stage rule out the possibility that nonhomologous end joining is also aberrant, as is the case in human cells lacking functional WRN (Chen *et al*., 2003; Otsuki *et al*., 2007), since such end joining is unlikely to yield scoreable clones in the assays used here.

Mechanistically, it has been difficult to differentiate between the impact of the exonuclease and helicase activities of WRN *in vivo* since RNAi ablates all activities, while point mutants may act as dominant negatives (Crabbe *et al*., 2004). By studying a model organism in which the WRN exonuclease activity is encoded on a genomic locus distinct from any putative partner helicase, we can readily ablate the exonuclease activity without the possibility of creating dominant negative complexes. Our data presented here clearly demonstrate the importance of WRN exonuclease in restraining mitotic recombination. Furthermore, it is likely that at least some of the recombination detected in the *DmWRNexo* mutants occurs as a result of deficiencies in resolving aberrant DNA structures arising during DNA replication. The observed hypersensitivity of *CG7670^e04496^* homozygotes to CPT is indicative of a role for DmWRNexo at collapsed replication forks, as predicted from human studies (Pichierri *et al*., 2001; Rodriguez-Lopez *et al*., 2002, 2007; Pirzio *et al*., 2008). Human WRN can regress replication forks *in vitro* (Machwe *et al*., 2006); how it supports re-establishment of collapsed forks *in vivo* is less clear, but our data suggest that the exonuclease activity of WRN may be important in preventing hyper-recombination at this stage. We speculate that DmWRNexo, like human WRN (Saintigny *et al*., 2002; Dhillon *et al*., 2007; Rodriguez-Lopez *et al*., 2007), may act to prevent Holliday junction accumulation at stalled or collapsed replication forks, and that DNA end processing activity of the exonuclease (Perry *et al*., 2006) may be critical to direct fork re-establishment. Such end processing could result in removal of DNA strands that would otherwise be used in the strand invasion step in homologous recombination. Thus, in cells lacking DmWRNexo, collapsed replication forks (such as at CPT-induced breaks) would persist, and promote Holliday junction formation and homologous recombination.

This study demonstrates the strength of using a genetically amenable model system for analysis of genes associated with genomic instability and human aging, even though the adult is largely postmitotic; absence of DmWRNexo function through fly development manifests as a hyper-recombinant phenotype in the mature adult. This raises the exciting possibility of using the short-lived fruit fly as model system for analysis and experimental modulation of WRN function *in vivo*.

## Experimental procedures

### Bioinformatics

BLAST searches (Altschul *et al*., 1997) were conducted against Release 4.0 of the *Drosophila melanogaster* genome sequence, and reciprocally against the human protein RefSeq database. BioEdit was used to generate the alignments following processing with Clustal W. Structural predictions were carried out using SWISS-MODEL (Peitsch, 1996; Schwede *et al*., 2003) based on the structure of human WRN exonuclease domain (Perry *et al*., 2006).

### DNA and RNA analysis

Total RNA was extracted from flies using RNeasy spin columns (Qiagen, Crawley, West Sussex, UK) and quantitated using a Qubit fluorometer (Invitrogen, Paisley, UK). For analysis of transcript levels, one-step RT-PCR (Qiagen) was carried out with gene-specific primers (CG7670 Exon1–2 F: 5′-ATGAAGTTCCCAAGGAAGAGG-3′; CG7670 Exon1–2 R: 5′-GATGGCGGCGTACATTAGTT-3′; actin 5C F: 5′-CACCGGTATCGTTCTGGACT-3′, actin 5C R: 5′-GGACTCGTCGTACTCCTGCT-3′) using 0.5 µg total RNA. Products were analysed on 0.9% agarose gels stained with ethidium bromide, against a 100-bp ladder (Roche, Burgess Hill, West Sussex, UK).

To clone *CG7670*, cDNA was prepared from freshly isolated RNA from female flies (TM6B/TM3, wild type for *CG7670*) using Omniscript reverse transcriptase (Qiagen) and random hexamer primers (Operon, Cologne, Germany) or oligo-dt(16) primers (Applied Biosystems, Warrington, UK). The cDNA was PCR-amplified using pfx50 proofreading DNA polymerase (Invitrogen) and primers Forward F1A (CGGGTTATGGAAAAATATTTAACAAAAATGCCC) and Reverse R-2 A (AGCTTACAGAGTCACCTCGTTGATCTTGG), to yield a blunt-end PCR product which was cloned into TOPO vector (Zero Blunt®TOPO® PCR Cloning Kit, Invitrogen). DNA sequencing was performed in-house by Geneservice on an ABI 3730xl DNA Analyser.

### Fly stocks

Fly stocks were obtained from the Bloomington *Drosophila* Stock Center (http://flystocks.bio.indiana.edu/), and were maintained on a standard oatmeal, yeast, molasses and agar medium. Wing blades were dissected from flies stored in 70% ethanol, mounted in Gary's Magic Mountant (Lawrence *et al*., 1986) and analysed by brightfield microscopy.

### Camptothecin sensitivity studies

Fly medium containing 60 g L^−1^ each of dextrose and yeast, 3% w/v nipagin and 3% v/v propionic acid was supplemented with CPT in a 5% ethanol/5% Tween-20 solution to achieve final CPT concentrations of 0–0.8 µm in vials containing 10 mL fly food (Cunhe *et al.*, 2002). Heterozygous *CG7670^e04496^/TM6B* flies were crossed and eggs were seeded into 4–5 vials per dose at ~200 eggs/vial, according to Clancy & Kennington (2001) and allowed to develop at 18 °C. Surviving heterozygous and homozygous adult flies were scored.
